# Trends in COVID-19 patient characteristics in a large electronic health record database in the United States: A cohort study

**DOI:** 10.1371/journal.pone.0271501

**Published:** 2022-07-20

**Authors:** Caihua Liang, Rachel P. Ogilvie, Michael Doherty, C. Robin Clifford, Andrea K. Chomistek, Robert Gately, Jennifer Song, Cheryl Enger, John Seeger, Nancy D. Lin, Florence T. Wang

**Affiliations:** 1 Optum Epidemiology, Boston, MA, United States of America; 2 IQVIA, King of Prussia, PA, United States of America; Shahid Beheshti University of Medical Sciences, ISLAMIC REPUBLIC OF IRAN

## Abstract

**Background:**

Electronic health record (EHR) databases provide an opportunity to facilitate characterization and trends in patients with COVID-19.

**Methods:**

Patients with COVID-19 were identified based on an ICD-10 diagnosis code for COVID-19 (U07.1) and/or a positive SARS-CoV-2 viral lab result from January 2020 to November 2020. Patients were characterized in terms of demographics, healthcare utilization, clinical comorbidities, therapies, laboratory results, and procedures/care received, including critical care, intubation/ventilation, and occurrence of death were described, overall and by month.

**Results:**

There were 393,773 patients with COVID-19 and 56,996 with a COVID-19 associated hospitalization. A greater percentage of patients hospitalized with COVID-19 relative to all COVID-19 cases were older, male, African American, and lived in the Northeast and South. The most common comorbidities before admission/infection date were hypertension (40.8%), diabetes (29.5%), and obesity (23.8%), and the most common diagnoses during hospitalization were pneumonia (59.6%), acute respiratory failure (44.8%), and dyspnea (28.0%). A total of 85.7% of patients hospitalized with COVID-19 had CRP values > 10 mg/L, 75.5% had fibrinogen values > 400 mg/dL, and 76.8% had D-dimer values > 250 ng/mL. Median values for platelets, CRP, lactate dehydrogenase, D-dimer, and fibrinogen tended to decrease from January-March to November. The use of chloroquine/hydroxychloroquine during hospitalization peaked by March (71.2%) and was used rarely by May (5.1%) and less than 1% afterwards, while the use of remdesivir had increased by May (10.0%) followed by dexamethasone by June (27.7%). All-cause mortality was 3.2% overall and 15.0% among those hospitalized; 21.0% received critical care and 16.0% received intubation/ventilation/ECMO.

**Conclusions:**

This study characterizes US patients with COVID-19 and their management during hospitalization over the first eleven months of this disease pandemic.

## Introduction

Severe Acute Respiratory Syndrome coronavirus 2 (SARS-COV-2) was first reported in Wuhan, China at the end of 2019 [1]. On March 11, 2020, the World Health Organization (WHO) declared coronavirus disease 2019 (COVID-19), the disease that SARS-COV-2 causes, a pandemic. As of June 28, 2021, there were approximately 181 million confirmed cases and 3.9 million deaths worldwide, including approximately 33.6 million confirmed cases and 604,000 deaths in the United States (US) [2].

As a novel disease, COVID-19 requires an extensive description of patients’ characteristics and correlates, along with information on management and outcomes to inform prevention and treatment strategies, especially among immunocompromised patients such as those with cancer [3]. Much of the existing description is in the form of case reports or case series, collected at a single site or hospital system, or one time point [4, 5]. To address the limited availability of large-scale, longitudinal, geographically diverse information on patients with COVID-19, Optum has developed a large electronic health record (EHR) database sourced from providers actively diagnosing and treating patients with COVID-19 throughout the US. This centralized EHR database of patients with COVID-19 provides a source within which to characterize patients with COVID-19 and evaluate their clinical outcomes, along with time trends in the first year of the pandemic.

## Materials and methods

### Study design, setting, and participants

This study was sourced from patients with COVID-19 between January 2020 and November 2020. The subset of patients with COVID-19 who were hospitalized during the same period was also identified. The presence of COVID-19 infection was based on a record of a specific International Classification of Diseases 10 (ICD-10) diagnosis code for SARS-COV-2 (U07.1) and/or a positive SARS-COV-2 viral test, which included real-time reverse transcription-polymerase chain reaction (RT-PCR) and nucleic acid amplification tests (NAATs) SARS-CoV-2 tests. Patients with serologic antibody tests only (i.e., no record of a confirmatory COVID-19 diagnosis code nor a positive SARS-CoV-2 viral laboratory test result) were not included. The infection date was set as the earlier date of diagnosis and positive lab test results, while the cohort entry date for the hospitalized patients was set to the later of infection date or hospital admission, as some patients were already hospitalized when the diagnosis of infection was made, and some patients were admitted to the hospital after being diagnosed. Setting the cohort entry date in this way allows for the description of patients’ clinical characteristics at the time of hospitalization with COVID-19. The presence of comorbidities and concomitant medication use were assessed in the 21 days before and including the cohort entry date. Patient characteristics were captured from cohort entry to the earlier of the discharge date or 30 days after cohort entry.

### Variables

All variables in this study were taken from the structured EHR data and were code-based. Patient demographics were derived from the EHR at the time of cohort entry (age in years, sex [male/female], race [African American/Asian/White/Other or Unknown], ethnicity [Hispanic/Not Hispanic/Unknown], region [Northeast/Midwest/South/West/Other or Unknown]). Comorbidities (diabetes, obesity, COPD, asthma, hypertension, coronary artery disease, congestive heart failure, liver disease, cancer) were assessed based on ICD-10 codes in the 21 days before or on the cohort entry date and patient-reported medication use (statins, angiotensin-converting enzyme inhibitors/angiotensin receptor blockers [ACEs/ARBs], nonsteroidal anti-inflammatory drugs [NSAIDs], corticosteroids for systemic use and proton pump inhibitors [PPIs]) was assessed via Anatomic Therapeutic Chemical (ATC) codes in the 21 days before and including the cohort entry date. For those hospitalized, information was collected on the duration of the hospitalization, vital signs (temperature, oxygen saturation), and laboratory tests and biomarkers (platelet count, C-reactive protein [CRP], ferritin, total lactate dehydrogenase, D-dimer, fibrinogen) via the initial occurrence of Logical Observation Identifiers Names and Codes (LOINCs) on or after hospital admission. Presenting symptoms or complications (hypoxemia, fever, cough, nausea/vomiting, malaise and fatigue, dyspnea/shortness of breath, acute respiratory failure, pneumonia, sepsis, coagulation defects, or hemorrhagic conditions, arrhythmia, heart failure, myocardial infarction) during hospitalization were assessed via ICD-10 codes. Medications administered (chloroquine/hydroxychloroquine, lopinavir/ritonavir, remdesivir, dexamethasone, ACEs/ARBs, anticoagulants, immunosuppressants, antibacterials for systemic use, antivirals for systemic use, and corticosteroids for systemic use) were assessed via ATC and Optum proprietary codes. During follow-up, mechanical ventilation (including intubation/ventilation/extracorporeal membrane oxygenation) and critical care were ascertained using Current Procedure Terminology (CPT^®^) and ICD-10 procedure codes during hospitalization. Mortality was determined via linkage to the Social Security Administration’s Death Master File or as indicated within the medical record. Data lag for the Death Master File is approximately 6–9 months while the lag for death indicated in the medical record is approximately 2 months.

### Data source

Given the importance of describing the clinical course of infection with COVID-19, Optum developed a low latency data pipeline that balances shortened data lag with the completeness of clinical information. The patients in this study were identified from Optum’s EHR Database derived from the electronic health records of a network of healthcare provider organizations across the United States that include more than 700 hospitals and 7000 clinics. A total of 48% of the contributing electronic medical record (EMR)/EHR systems are from the Midwest, 20% from the South, 20% from the Northeast, and 8% from the West. This database incorporates clinical and medical administrative data from both inpatient and ambulatory EMRs, practice management systems, and numerous other internal systems. The data are processed from across the continuum of care, including acute inpatient stays and outpatient visits, and are incorporated into the database on a biweekly basis, representing a near-time capture of information. Optum’s COVID-19 EHR database captures diagnostics specific to the COVID-19 patient during initial presentation, acute illness, and convalescence with over 500 mapped labs and bedside observations, including COVID-19 specific testing. The database is certified as de-identified by an independent statistical expert following Health Insurance Portability and Accountability Act (HIPAA) statistical de-identification rules and managed according to Optum customer data use agreements. The database was deidentified before the authors gained access. Data were extracted on December 10, 2020.

### Statistical methods

Frequencies and percentages were reported for binary and categorical variables, while medians and interquartile ranges (IQRs) were reported for continuous variables. Characterizations were described and visualized overall and by cohort entry month or week. All analyses were conducted in SAS 9.4 (SAS Institute Inc. Cary, NC). Strengthening the Reporting of Observational studies in Epidemiology (STROBE) guidelines were followed in the reporting of this research [6].

## Results

**Fig 1:**
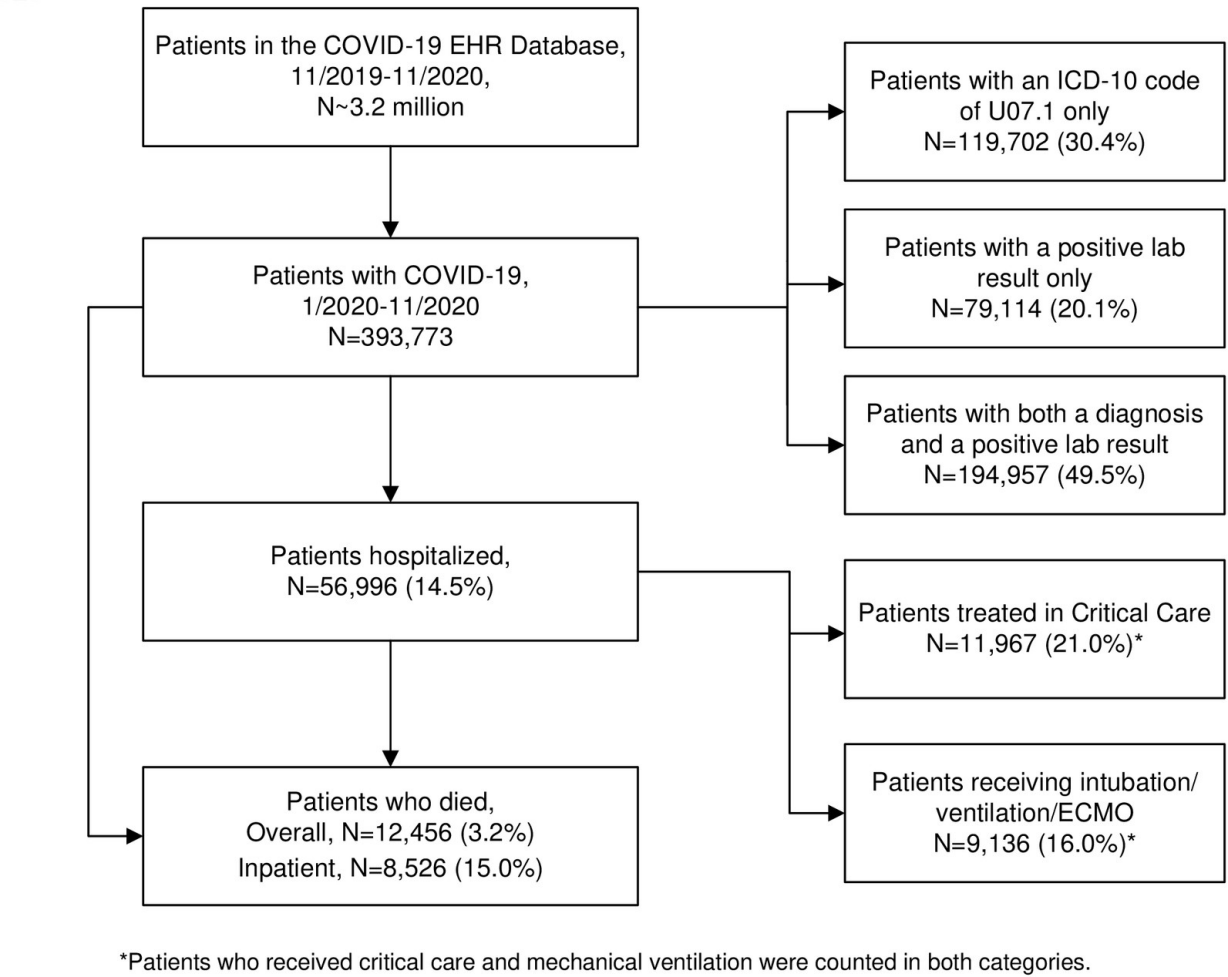
Flowchart of diagnosed COVID-19 cases: The Optum EHR COVID-19 database January-November 2020. *Patients who received critical care mechanical ventilation were counted in both categories.

The flow chart for the identification of COVID-19 cases within the Optum COVID-19 EHR database is shown in Fig 1. Patients identified to have COVID-19 were 393,733 out of the approximately 3.2 million included in the database with a COVID-19 related code Among these patients, 49.5% (N = 194,957) were identified with both a diagnosis and a positive lab result, 30.4% (N = 119,702) with a diagnostic code only, 20.1% (N = 79,114) with a positive lab result only. A total of 56,996 patients (14.5%) were hospitalized, including 11,967 (21.0%) who received critical care and 9,136 (16.0%) treated with mechanical ventilation. There were 12,456 deaths among all identified cases of COVID-19, corresponding to an overall mortality of 3.2%, and 8,526 deaths among patients hospitalized with COVID-19, corresponding to a mortality of 15.0%.

**Fig 2:**
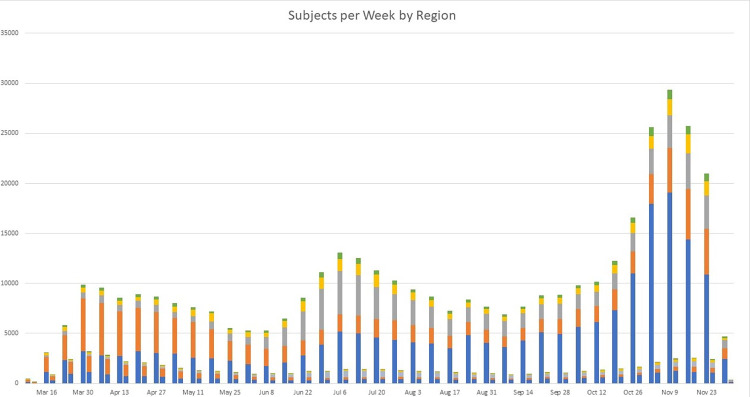


Basic demographics for patients with COVID-19 and the subset of patients hospitalized are displayed in Fig 2. A greater percentage of patients hospitalized with COVID-19 relative to all COVID-19 cases were older (35.0% aged 70 years and older among hospitalized vs. 13.8% among all cases), male (49.5% vs. 44.6%), and African American (20.5% vs. 12.9%).

**Fig 3:**
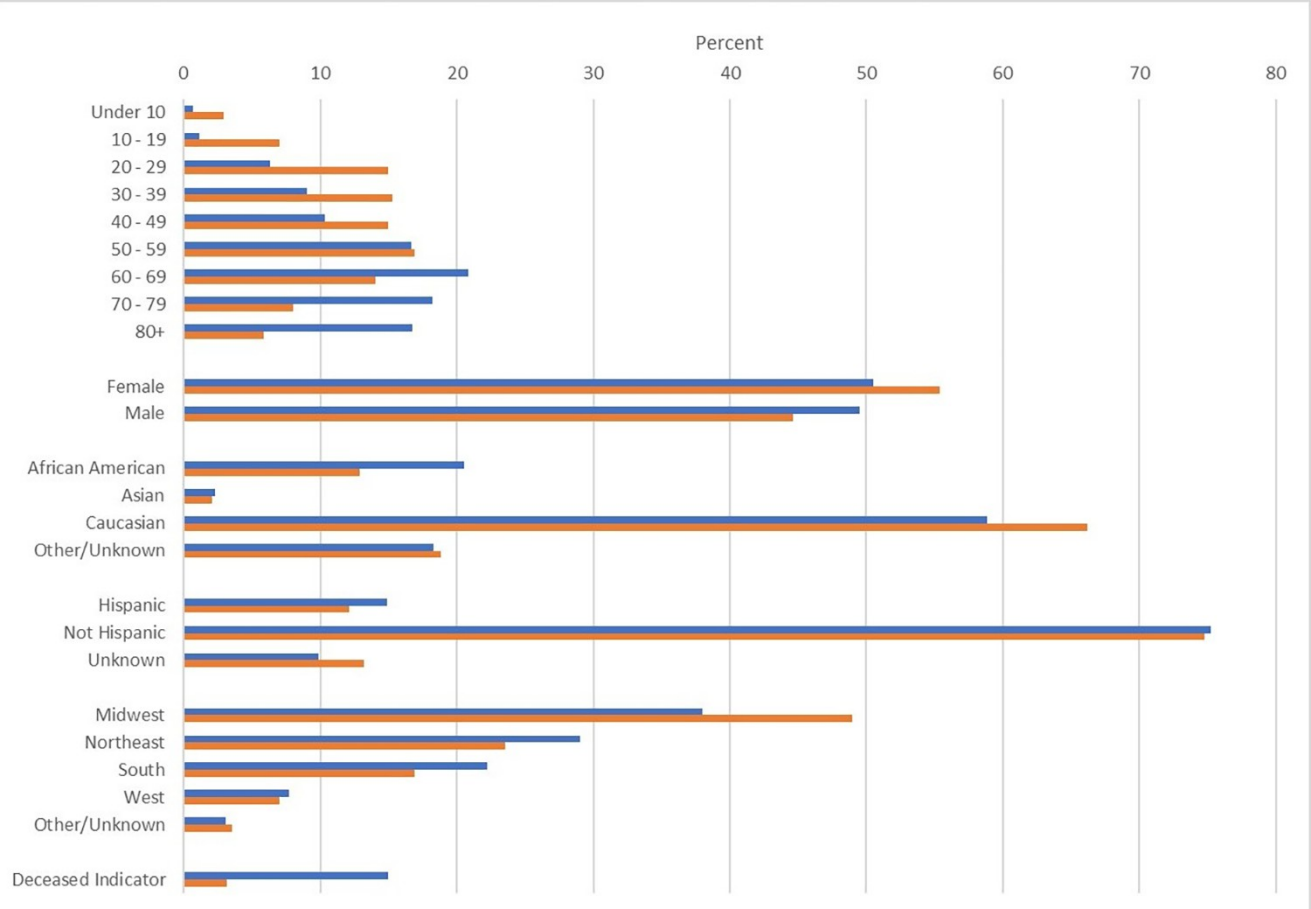


Fig 3 demonstrates the trend and distribution of COVID-19 cases biweekly by region in the left bar of each pair. There were three peaks of case counts observed; the first occurred in late- March to April, the second in late -June to July, and the third and largest in early November. A greater percentage of patients were from the Northeast in March, April, and May, while in June through November, the most patients were from the Midwest.

The trend and distribution of patients hospitalized with COVID-19 were also visualized in the right bar of each pair in Fig 3. The number of hospitalized cases peaked in early April when the majority were from the Northeast. In the summer months, hospitalized cases declined overall though a larger percentage were from the South. Hospitalized cases grew again in the fall, with the largest percentage from the Midwest.

Comorbidities and patient-reported medication use among those hospitalized with COVID-19 are shown in Table 1. The most common insurance type was multiple types at 28.5%, followed by commercial only at 27.7%. The majority (76.7%) of patients presented to the emergency department before hospital admission. A total of 26.0% of the hospitalized patients had a diagnostic code only as their cohort qualifying event, 3.8% had a lab result only, and 70.2% had both. The most common comorbidities were hypertension (40.8%), diabetes (29.5%), and obesity (23.8%), while the most common patient-reported medication used was statins (26.2%).

**Table 1:** Demographics and comorbidities among hospitalized patients with COVID-19^a^.

	No. (%)
**Total N**	56,996 (100.0)
**Cohort qualifying event**	
Diagnosis code and lab result detected	40,008 (70.2)
Diagnosis code only	14,822 (26.0)
Lab results detected only	2,166 (3.8)
**Comorbidities**	
Diabetes	16,826 (29.5)
Obesity	13,544 (23.8)
Pulmonary disease	
COPD	5,830 (10.2)
Asthma	4,948 (8.7)
Cardiovascular disease	
Hypertension	23,229 (40.8)
Coronary artery disease	8,407 (14.8)
Congestive heart failure	7,242 (12.7)
Liver disease	2,671 (4.7)
Cancer	4,369 (7.7)
**Patient-reported medication use**	
Statins	14,933 (26.2)
ACEs/ARBs	11,517 (20.2)
NSAIDS	5,908 (10.4)
Corticosteroids for systemic use	6,827 (12.0)
PPIs	9,966 (17.5)
**Healthcare encounter settings 21 days before and including cohort entry date**	
Inpatient	4,684 (8.2)
Outpatient	10,479 (18.4)
Emergency department	43,724 (76.7)
**Insurance type on admission date**	
Multiple	16,266 (28.5)
Commercial	15,775 (27.7)
Medicare	12,364 (21.7)
Medicaid	5,111 (9.0)
Other payer type	2,936 (5.2)
Uninsured	973 (1.7)
Unknown	3,571 (6.3)

Abbreviations: COPD, chronic obstructive pulmonary disease; ACE, angiotensin-converting enzyme; ARB, angiotensin II receptor blocker; NSAIDs, non-steroidal anti-inflammatory drugs; PPIs, proton-pump inhibitors

^a^ Demographics assessed on admission date, comorbidities, and medications assessed in the 21 days before and including the admission date.

Characterization of the hospitalized population during the hospitalization both overall and by admission month is presented in Table 2. From January through March, men and African Americans made up a larger percentage of patients hospitalized with COVID-19, while by the summer the hospitalized population was younger, more female, and more Hispanic. By November, the median age of the patients hospitalized with COVID-19 peaked at 65 (interquartile range [IQR]: 52,77) and most patients were white and non-Hispanic. The median length of hospitalization declined from 8 days (IQR: 5, 16) in January through March to 6 days (IQR:4, 9) in November.

**Table 2:** Characterization of patients hospitalized with COVID-19 during hospitalization overall* and by admission month in 2020.

	Total	January to March	April	May	June	July	August	September	October	November
**Age in years (median, IQR)**	62 (47, 75)	63 (51, 73)	63 (51, 76)	60 (44, 74)	57 (40, 70)	60 (44, 73)	61 (42, 74)	61 (42, 74)	64 (48, 76)	65 (52, 77)
**Gender (N, %)**										
Female	28,782 (50.5)	1,873 (41.6)	4,883 (46.9)	3,030 (51.7)	2,471 (53.6)	3,176 (52.2)	2,654 (52.9)	2,338 (54.1)	3,251 (53.9)	5,106 (50.2)
Male	28,214 (49.5)	2,624 (58.4)	5,539 (53.1)	2,829 (48.3)	2,140 (46.4)	2,906 (47.8)	2,359 (47.1)	1,980 (45.9)	2,777 (46.1)	5,060 (49.8)
**Race (N, %)**										
African American	11,694 (20.5)	1,359 (30.2)	2,783 (26.7)	1,439 (24.6)	945 (20.5)	1,483 (24.4)	891 (17.8)	635 (14.7)	791 (13.1)	1,368 (13.5)
Asian	1,336 (2.3)	161 (3.6)	393 (3.8)	161 (2.7)	110 (2.4)	108 (1.8)	89 (1.8)	83 (1.9)	100 (1.7)	131 (1.3)
White	33,524 (58.8)	2,111 (46.9)	5,058 (48.5)	3,033 (51.8)	2,566 (55.6)	3,427 (56.3)	3,142 (62.7)	2,846 (65.9)	4,269 (70.8)	7,072 (69.6)
Other/Unknown	10,442 (18.3)	866 (19.3)	2,188 (21.0)	1,226 (20.9)	990 (21.5)	1,064 (17.5)	891 (17.8)	754 (17.5)	868 (14.4)	1,595 (15.7)
**Ethnicity (N, %)**										
Hispanic	8,497 (14.9)	377 (8.4)	1,515 (14.5)	1,133 (19.3)	1,155 (25.0)	1,150 (18.9)	767 (15.3)	573 (13.3)	692 (11.5)	1,135 (11.2)
Not Hispanic	42,881 (75.2)	3,478 (77.3)	7,747 (74.3)	4,239 (72.4)	3,073 (66.6)	4,390 (72.2)	3,755 (74.9)	3,358 (77.8)	4,811 (79.8)	8,030 (79.0)
Unknown	5,618 (9.9)	642 (14.3)	1,160 (11.1)	487 (8.3)	383 (8.3)	542 (8.9)	491 (9.8)	387 (9.0)	525 (8.7)	1,001 (9.8)
**Region (N, %)**										
Northeast	16,548 (29.0)	2,236 (49.7)	5,253 (50.4)	2,457 (41.9)	1,223 (26.5)	854 (14.0)	787 (15.7)	796 (18.4)	1,056 (17.5)	1,886 (18.6)
Midwest	21,647 (38.0)	1,639 (36.4)	3,525 (33.8)	2,130 (36.4)	1,478 (32.1)	1,796 (29.5)	1,851 (36.9)	1,694 (39.2)	2,722 (45.2)	4,812 (47.3)
South	12,654 (22.2)	247 (5.5)	777 (7.5)	746 (12.7)	1,418 (30.8)	2,758 (45.3)	1,820 (36.3)	1,309 (30.3)	1,473 (24.4)	2,106 (20.7)
West	4,378 (7.7)	274 (6.1)	569 (5.5)	336 (5.7)	331 (7.2)	456 (7.5)	412 (8.2)	380 (8.8)	586 (9.7)	1,034 (10.2)
Other/Unknown	1,769 (3.1)	101 (2.2)	298 (2.9)	190 (3.2)	161 (3.5)	218 (3.6)	143 (2.9)	139 (3.2)	191 (3.2)	328 (3.2)
**Duration of Hospital Stay in Days (median, IQR)**	6 (4.0, 11.0)	8 (5.0, 16.0)	7 (4.0, 12.0)	6 (3.0, 11.0)	5 (3.0, 9.0)	6 (4.0, 11.0)	6 (4.0, 10.0)	6 (4.0, 10.0)	6 (4.0, 10.0)	6 (4.0, 9.0)
**Observations** *										
Temperature ^°^C (median, IQR)	36.8 (36.5, 37.1)	36.9 (36.6, 37.3)	36.8 (36.5, 37.1)	36.8 (36.5, 37.1)	36.8 (36.5, 37.2)	36.8 (36.5, 37.2)	36.7 (36.5, 37.1)	36.7 (36.4, 37.0)	36.7 (36.4, 37.0)	36.7 (36.4, 37.0)
> 38° (N, %)	3,609 (6.6)	436 (10.2)	740 (7.4)	348 (6.3)	355 (8.0)	501 (8.6)	275 (5.7)	180 (4.3)	280 (4.8)	494 (5.0)
Oxygen saturation (SpO2), % (median, IQR)	95 (93.0, 97.3)	95.3 (93.0, 97.4)	95.2 (92.6, 97.3)	95.7 (93.0, 97.4)	95.2 (93.0, 97.3)	95 (93.0, 97.9)	95.5 (93.0, 98.0)	95 (93.0, 98.0)	95 (93.0, 97.3)	95 (92.0, 97.0)
< 90% (N, %)	2,457 (10.1)	249 (12.3)	456 (12.1)	215 (10.2)	169 (8.2)	288 (8.6)	184 (7.7)	186 (9.6)	259 (9.0)	451 (12.3)
Platelet count (PLT) x 10^9^ per L (median, IQR)	224 (169.0, 298.0)	219 (163.0, 299.0)	233 (172.0, 313.0)	227 (170.0, 304.0)	226 (169.0, 297.0)	226 (172.0, 302.0)	225 (169.0, 292.0)	220 (169.0, 289.0)	220 (167.0, 289.0)	220 (167.0, 291.0)
< 150 (N, %)	9,355 (17.0)	827 (18.9)	1,639 (16.3)	942 (16.8)	734 (17.0)	935 (15.7)	825 (17.1)	715 (17.0)	1,047 (18.0)	1,691 (17.3)
C-reactive protein (CRP), mg/L (median, IQR)	60.5 (21.1, 124.0)	81.6 (34.9, 151.4)	71 (26.2, 137.0)	61 (18.0, 129.2)	55 (17.0, 117.0)	57.9 (20.4, 118.0)	51.6 (18.0, 108.0)	51.7 (18.0, 109.0)	51 (17.6, 108.0)	53 (19.4, 109.0)
> 10 (N, %)	30,979 (85.7)	3,235 (91.5)	7,473 (88.1)	3,219 (82.3)	2,183 (82.6)	3,052 (84.7)	2,245 (84.1)	1,804 (83.6)	2,676 (83.6)	5,092 (85.9)
Ferritin, ng/mL (median, IQR)	478 (219.1, 942.0)	636 (323.1, 1214.3)	518 (245.0, 1018.0)	408 (165.0, 856.0)	426 (178.0, 873.3)	466.6 (216.9, 960.1)	453.6 (197.6, 891.3)	446.7 (204.2, 878.2)	436.4 (211.0, 847.7)	459.9 (221.0, 875.0)
> 300 (N, %)	22,216 (66.3)	2,494 (77.2)	5,417 (69.0)	2,210 (59.9)	1,485 (61.7)	2,347 (65.8)	1,671 (63.4)	1,299 (65.3)	1,906 (64.0)	3,387 (65.7)
Lactate dehydrogenase. Total (LDH), U/L (median, IQR)	310 (232.0, 428.0)	342 (260.0, 466.0)	322 (239.0, 446.0)	287 (213.0, 408.0)	302.5 (224.0, 431.0)	325 (241.0, 443.0)	304 (226.0, 425.0)	297.5 (223.0, 413.0)	297 (223.0, 400.0)	297 (227.0, 398.0)
> 280 (N, %)	19,726 (58.9)	2,236 (68.7)	4,858 (62.1)	1,844 (52.2)	1,393 (57.3)	2,198 (62.5)	1,451 (56.9)	1,130 (55.4)	1,620 (54.8)	2,996 (55.9)
D-Dimer, ng/mL (median, IQR)	465 (264.0, 880.0)	490 (285.0, 925.0)	489 (274.5, 925.0)	462 (257.0, 844.5)	420 (242.3, 795.0)	447.5 (255.0, 854.0)	472.8 (275.0, 910.0)	475 (262.0, 910.0)	460 (260.0, 905.8)	440 (256.0, 825.0)
> 250 (N, %)	19,021 (76.8)	1,975 (80.1)	4,557 (77.8)	1,728 (75.8)	1,267 (73.8)	1,711 (75.6)	1,470 (78.6)	1,140 (75.7)	1,680 (76.2)	3,493 (75.8)
Fibrinogen, mg/dL (median, IQR)	529 (403.0, 667.0)	626 (472.5, 700.0)	552 (410.0, 700.0)	515 (389.0, 656.0)	513 (390.0, 646.0)	535 (419.0, 660.0)	507 (386.0, 646.0)	496 (384.0, 624.0)	513.5 (397.0, 633.5)	516 (403.0, 639.5)
> 400 (N, %)	12,116 (75.5)	955 (83.5)	2,543 (76.7)	1,430 (73.2)	958 (72.7)	1,571 (77.8)	975 (72.1)	780 (72.0)	1,099 (74.3)	1,805 (75.7)
Diagnosis* **(N, %)**										
Hypoxemia	14,718 (25.8)	1,669 (37.1)	3,736 (35.8)	1,376 (23.5)	900 (19.5)	1,527 (25.1)	1,007 (20.1)	829 (19.2)	1,273 (21.1)	2,401 (23.6)
Fever	9,158 (16.1)	1,860 (41.4)	2,609 (25.0)	988 (16.9)	656 (14.2)	884 (14.5)	545 (10.9)	382 (8.8)	516 (8.6)	718 (7.1)
Cough	7,182 (12.6)	1,686 (37.5)	2,388 (22.9)	623 (10.6)	385 (8.3)	593 (9.8)	322 (6.4)	254 (5.9)	368 (6.1)	563 (5.5)
Nausea/vomiting	3,711 (6.5)	389 (8.7)	788 (7.6)	472 (8.1)	341 (7.4)	428 (7.0)	274 (5.5)	275 (6.4)	326 (5.4)	418 (4.1)
Malaise and fatigue	7,483 (13.1)	815 (18.1)	1,724 (16.5)	845 (14.4)	502 (10.9)	696 (11.4)	517 (10.3)	493 (11.4)	736 (12.2)	1,155 (11.4)
Dyspnea/Shortness of Breath	15,979 (28.0)	2,300 (51.1)	4,656 (44.7)	1,804 (30.8)	1,108 (24.0)	1,339 (22.0)	950 (19.0)	782 (18.1)	1,161 (19.3)	1,879 (18.5)
Acute Respiratory Failure	25,525 (44.8)	2,962 (65.9)	6,235 (59.8)	2,531 (43.2)	1,622 (35.2)	2,457 (40.4)	1,832 (36.5)	1,570 (36.4)	2,375 (39.4)	3,941 (38.8)
Pneumonia	33,946 (59.6)	3,957 (88.0)	8,319 (79.8)	3,560 (60.8)	2,241 (48.6)	3,053 (50.2)	2,367 (47.2)	1,989 (46.1)	3,112 (51.6)	5,348 (52.6)
Sepsis	12,646 (22.2)	1,929 (42.9)	3,435 (33.0)	1,413 (24.1)	790 (17.1)	1,069 (17.6)	843 (16.8)	790 (18.3)	1,038 (17.2)	1,339 (13.2)
Coagulation defects or hemorrhagic conditions	3,490 (6.1)	397 (8.8)	1,186 (11.4)	466 (8.0)	236 (5.1)	272 (4.5)	214 (4.3)	226 (5.2)	242 (4.0)	251 (2.5)
Arrhythmia	4,799 (8.4)	701 (15.6)	1,413 (13.6)	513 (8.8)	297 (6.4)	401 (6.6)	343 (6.8)	322 (7.5)	390 (6.5)	419 (4.1)
Heart failure	7,934 (13.9)	645 (14.3)	1,788 (17.2)	1,007 (17.2)	661 (14.3)	709 (11.7)	576 (11.5)	566 (13.1)	866 (14.4)	1,116 (11.0)
MI	3,437 (6.0)	481 (10.7)	1,011 (9.7)	369 (6.3)	219 (4.7)	260 (4.3)	225 (4.5)	184 (4.3)	305 (5.1)	383 (3.8)
Treatment* **(N, %)**										
Chloroquine/ hydroxychloroquine	8,852 (15.5)	3,203 (71.2)	5,036 (48.3)	299 (5.1)	59 (1.3)	87 (1.4)	39 (0.8)	24 (0.6)	45 (0.7)	60 (0.6)
Lopinavir/ritonavir	427 (0.7)	241 (5.4)	171 (1.6)	4 (0.1)	4 (0.1)	3 (0.0)	2 (0.0)	1 (0.0)	1 (0.0)	0 (0.0)
Remdesivir	12,990 (22.8)	65 (1.4)	231 (2.2)	586 (10.0)	730 (15.8)	1,680 (27.6)	1,373 (27.4)	1,310 (30.3)	2,254 (37.4)	4,761 (46.8)
Dexamethasone	16,698 (29.3)	195 (4.3)	538 (5.2)	420 (7.2)	1,279 (27.7)	2,672 (43.9)	2,118 (42.3)	1,772 (41.0)	2,766 (45.9)	4,938 (48.6)
ACEs/ARBs	12,313 (21.6)	882 (19.6)	2,030 (19.5)	1,134 (19.4)	932 (20.2)	1,285 (21.1)	1,092 (21.8)	953 (22.1)	1,460 (24.2)	2,545 (25.0)
Anticoagulants	45,509 (79.8)	4,058 (90.2)	9,289 (89.1)	4,699 (80.2)	3,708 (80.4)	4,540 (74.6)	3,623 (72.3)	3,152 (73.0)	4,535 (75.2)	7,905 (77.8)
Immunosuppressants	3,009 (5.3)	540 (12.0)	1,002 (9.6)	355 (6.1)	191 (4.1)	230 (3.8)	125 (2.5)	112 (2.6)	157 (2.6)	297 (2.9)
Systemic antibacterials	38,050 (66.8)	3,903 (86.8)	8,017 (76.9)	3,891 (66.4)	3,093 (67.1)	3,751 (61.7)	3,138 (62.6)	2,738 (63.4)	3,665 (60.8)	5,854 (57.6)
Systemic antivirals	1,847 (3.2)	426 (9.5)	549 (5.3)	160 (2.7)	100 (2.2)	130 (2.1)	102 (2.0)	92 (2.1)	123 (2.0)	165 (1.6)
Systemic corticosteroids	26,615 (46.7)	1,487 (33.1)	3,667 (35.2)	1,667 (28.5)	1,908 (41.4)	3,262 (53.6)	2,667 (53.2)	2,230 (51.6)	3,417 (56.7)	6,310 (62.1)
Outcomes* **(N, %)**										
Critical Care	11,967 (21.0)	1,389 (30.9)	2,924 (28.1)	1,472 (25.1)	908 (19.7)	1,136 (18.7)	856 (17.1)	824 (19.1)	1,102 (18.3)	1,356 (13.3)
Intubation/Ventilation/ECMO	9,136 (16.0)	1,397 (31.1)	2,205 (21.2)	994 (17.0)	594 (12.9)	840 (13.8)	644 (12.8)	575 (13.3)	858 (14.2)	1,029 (10.1)
Death	8,526 (15.0)	1,040 (23.1)	2,360 (22.6)	1,028 (17.5)	593 (12.9)	916 (15.1)	721 (14.4)	623 (14.4)	776 (12.9)	469 (4.6)

*All observations, diagnoses, treatments, and outcomes occurred during hospitalization

Abbreviations: MI, myocardial infarction, ACE, angiotensin-converting enzyme; ARB, angiotensin II receptor blocker; ICU, intensive care unit; ECMO, extracorporeal membrane oxygenation

Among hospitalized patients, 6.6% had a temperature > 38 degrees Celsius and 10.1% had an oxygen saturation < 90%. A total of 85.7% of patients hospitalized with COVID-19 had CRP values > 10 mg/L, 76.8% had D-dimer values > 250 ng/mL, and 75.5% had fibrinogen values > 400 mg/dL. Median values for platelets, CRP, lactate dehydrogenase, D-dimer, and fibrinogen tended to decrease from January-March to November. The most common presenting symptoms or complications during hospitalization were pneumonia (59.6%), followed by acute respiratory failure (44.8%), and dyspnea (28.0%). The prevalence of most symptoms and complications tended to decline from January through March to November.

The most common medications administered during hospitalization were anticoagulants (79.8%) followed by systemic antibacterials (66.8%) and corticosteroids (46.7%). The use of chloroquine/hydroxychloroquine declined throughout the study, with 71.2% of patients receiving it in January-March, 5.1% in May, and less than 1% from August to November. In contrast, remdesivir (1.4% in January-March vs. 46.8% in November) and dexamethasone (4.3% in January-March vs. 48.6% in November) increased during the same period. Use of lopinavir/ritonavir, anticoagulants, immunosuppressants, systemic antibacterials, and systemic antivirals declined from January to November.

The severity of disease and associated outcomes, as measured by receipt of critical care, mechanical ventilation, and occurrence of death, decreased over time. The proportion of patients receiving critical care during hospitalization dropped from 30.9% in January-March to 13.3% in November, receiving mechanical ventilation dropped from 31.1% to 10.1%, and those who died from 23.1% to 4.6%.

## Discussion

This characterization of US patients hospitalized with COVID-19 illustrates the clinical correlates and management of the disease over time in a large geographically diverse EHR database. The distribution of patients with COVID-19 and the subset who were hospitalized varied by age, region, race, and time. A change in treatment pattern was also observed, as the use of hydroxychloroquine decreased while the use of dexamethasone and remdesivir increased. Most presenting symptoms and complications as well as severe outcomes during hospitalization improved throughout the study period.

This EHR data source from which the patients with COVID-19 arose provides an opportunity to identify and characterize large numbers of patients with COVID-19, facilitating rapid observational assessments and providing insight into treatment and the outcome of infection. While randomized controlled trials continue to be seen as the gold standard to determine treatment efficacy for narrowly-defined hypotheses, real-world data sources provide useful insight into treatment effects during an evolving pandemic [7]. Because this database includes close to real-time data and is updated at least monthly, it can also serve as an important source of surveillance activities related to the COVID-19 pandemic in the US. Since it is larger than databases in previously published studies [8, 9], it may also have the power to detect rare correlates and outcomes.

In this database, the number of both cases of COVID-19 and hospitalized patients with COVID-19 peaked in early April and then declined until early July, when the number of cases had a second large peak while the number of hospitalized cases increased slightly. Both cases of COVID-19 and hospitalized patients with COVID-19 reached another peak in November. These peaks of cases were likely due to surges in different underlying regions and age groups over time. In March, hospitalized patients with COVID-19 were predominantly older and resided in the Northeast, with many outbreaks reported in nursing homes or assisting living facilities [10]. In contrast, as the pandemic continued into the summer, the median age of cases declined in the US and our data, and the median age peaked again in November [11]. Because the percentage of COVID-19 cases that were hospitalized increases with age [12], this may be a potential explanation for our findings. In addition, the differences observed between cases of COVID-19 and hospitalized patients with COVID-19 may reflect a lag between the timing of infection and the time of admission to the hospital. However, there was little difference between the admission date and the COVID-19 infection date. Consistent with previous studies [13], we also observed that a higher percentage of hospitalized patients were older, male, and African American compared with overall patients with COVID-19. The disproportionate burden of COVID-19 in these groups may indicate differences in underlying diseases, socioeconomic and occupational status, crowded housing, immune response, clinical features, and more compared with younger populations, females, and other races.

In line with other studies [13, 14], we observed that hypertension, diabetes, and obesity were common underlying comorbidities among patients with COVID-19, and these conditions may represent correlates of the demographic characteristics of affected patients. The prevalence of these conditions in our study was lower than those reported in previous studies. Reasons for this disparity include a broader region and a longer period of the pandemic covered in the analysis or under captured comorbidities. These reasons may also explain the relatively lower mortality rate in the overall population. The mortality rates in our study were 15% for hospitalized patients and 3.2% for overall patients, consistent with 15% for the hospitalized patients in the MMWR [15]. However, we observed a higher prevalence of severe outcomes than in previous reports; this could be explained by our application of using the broader category of critical care instead of ICU only and the broader category of mechanical ventilation instead of intubation, ventilation, or ECMO individually. We found that 21.0% of hospitalized patients received critical care and 16.0% received mechanical ventilation, in contrast to a previous report in which 14% of patients were treated in the ICU and 12% received invasive mechanical ventilation in the New York City area. In a study of patients readmitted for COVID-19, 15% were admitted to an ICU and 13% required invasive mechanical ventilation during their first admission [15].

The reduction of symptoms, conditions and severe outcomes among hospitalized patients over time aligns with the lessons and knowledge learned during this pandemic as treatment patterns were adjusted. We observed that the use of chloroquine/hydroxychloroquine peaked in January-March and dramatically dropped by May while the use of remdesivir and dexamethasone increased over the study period. This reflects the change in evidence and FDA emergency use authorizations for treatments throughout the pandemic. While hydroxychloroquine was originally touted as a promising treatment, recent randomized trials have demonstrated that this drug is no more effective than a placebo at preventing COVID-19 illness and is associated with more adverse events [16, 17]. In contrast, separate trials have shown the use of remdesivir is associated with lower median recovery time in hospitalized adults compared to placebo and the use of dexamethasone was associated with lower 28-day mortality among hospitalized patients with COVID-19 on respiratory support compared to usual care [18, 19]. This shows that treating clinicians are rapidly changing their prescribing patterns as additional data are released. The slight decline in the use of anticoagulants, immunosuppressants, antibacterials for systemic use, and antivirals for systemic use over time aligns with the improvement observed in inflammation-related biomarkers and coagulopathy profiles, corresponding to the decrease in severe outcomes over time. However, it is also possible that this decline is due in part to data lag.

This study has several limitations. While EHR data are valuable for the examination of clinical health care outcomes and treatment patterns, EHR databases have certain inherent limitations because the data are collected for clinical patient management, not research. The patient-reported medication use data are self-reported and do not indicate that medication was prescribed, filled, consumed or that it was taken as prescribed. However, for medications administered during hospitalization, underestimation is less likely. The presence of a diagnosis code may not always represent the presence of disease, as the diagnosis code may be incorrect or may represent a condition being evaluated or ruled out. Also, diagnoses and treatments received from providers outside of the network may not be recorded. Because the clinical information was recorded in the EHR during a stressful pandemic situation, it is possible that typical coding conventions were not followed [7]. Cases found in January and February may represent data errors instead of true cases. Additionally, some laboratory observations were not recorded for all patients. There may be a data lag in identifying hospitalizations, which would underestimate the number of hospitalized patients. In addition, although deaths were ascertained using all data available, underestimation is likely due to data lag. While the death indicator from the EHR does capture medically attended deaths, there may be a lag of up to 2 months for this field to be up to date. For medically unattended death, we relied on linkage to the Social Security Death Master File which has a lag of 6–9 months. Thus, deaths occurring outside of interaction with the EHR provider network may have been missed.

In conclusion, this study provides a characterization of US patients identified with COVID-19 and those hospitalized with COVID-19 and their management over the first 11 months of this disease outbreak. This characterization provides insight into the populations most affected by the disease and evidence for future observational research and may facilitate inferential study design.

## Supporting information

S1 Checklist STROBE statement. (DOC)

S1 Table Codes for defining variables in the Optum COVID-19 EHR database. (DOCX)
