# Supplementary material for: Trends in COVID-19 patient characteristics in a large electronic health record database in the United States: A cohort study
Source: PLoS One. 2022 Jul 20;17(7):e0271501. doi: 10.1371/journal.pone.0271501 (PMC9299343; doi:10.1371/journal.pone.0271501)
Supplement: S1 Table — (DOCX) [file pone.0271501.s002.docx]

**Supplemental Table 1: Codes for defining variables in the Optum COVID-19 EHR Database**

*CPT® copyright 2020 American Medical Association. All rights reserved.*

*Fee schedules, relative value units, conversion factors and/or related components are not assigned by the AMA, are not part of CPT, and the AMA is not recommending their use. The AMA does not directly or indirectly practice medicine or dispense medical services. The AMA assumes no liability for data contained or not contained herein.*

*CPT is a registered trademark of the American Medical Association.*

| **Diagnoses/Condition/Medication** | **Code type** | **Code** |
| --- | --- | --- |
| Type 2 diabetes | ICD-10 | E11* |
| Obesity | ICD-10 | E66* |
| COPD | ICD-10 | J44* |
| Asthma | ICD-10 | J45 |
| Hypertension | ICD-10 | I10 |
| Coronary artery disease | ICD-10 | I25* |
| Congestive heart failure | ICD-10 | I50* |
| Kidney disease | ICD-10 | N17*, N18*, N28*, I12*, I13* |
| Liver disease | ICD-10 | K70*-K77* |
| Cancer | ICD-10 | C00-D48 |
| Statins | ATC | C10AA* |
| ACEs/ARBs | ATC | C09AA*, C09CA* |
| NSAIDs | ATC | M01A* |
| PPIs | ATC | A02BC* |
| Temperature | LOINC | 8310-5 |
| Oxygen saturation | LOINC | 2708-6, 19211-2, 59408-5 |
| Platelet count | LOINC | 26515-7 |
| C-reactive protein | LOINC | 1988-5 |
| Ferritin | LOINC | 2776-4 |
| Lactase dehydrogenase | LOINC | 2532-0 |
| D-dimer | LOINC | 48065-7 |
| Fibrinogen | LOINC | 3255-7, 42772-4 |
| Fever | ICD-10 | R50.9, R50.81 |
| Cough | ICD-10 | R05 |
| Nausea/vomiting | ICD-10 | R11 |
| Malaise and fatigue | ICD-10 | R53* |
| Dyspnea or shortness of breath | ICD-10 | R06.00, R06.02, R06.09 |
| Acute respiratory failure | ICD-10 | J96.0* |
| Pneumonia | ICD-10 | J12*, J18*, J95.851 |
| Sepsis | ICD-10 | A41.9, A41.89, R65.21, R65.20 |
| Coagulation defects or hemorrhagic conditions | ICD-10 | D65, D68*, R79.1, |
| Arrhythmia | ICD-10 | I49* |
| Myocardial infarction | ICD-10 | I21* |
| Chloroquine/hydrocholorquine | ATC + Optum proprietary codes | P01BA* |
| Lopinavir/ritonavir | ATC + Optum proprietary codes | J05AR10 |
| Remdesivir | Optum proprietary codes |  |
| Dexamethasone | ATC | H02AB02 |
| ACEs/ARBs | ATC | C09AA*, C09CA* |
| Anticoagulants | ATC | B01A* |
| Immunosuppressants | ATC | L04A* |
| Antibacterials for systemic use | ATC | J01* |
| Antivirals for systemic use | ATC | J05* |
| Corticosteroids | ATC | H02A* |
| Critical care | CPT | Available upon request |
| ECMO | CPT and ICD-10 Procedure | 5A1522H, CPT codes available upon request |
| Intubation/ventilation | CPT and ICD-10 Procedure | 0BH17EZ, 0BH18EZ, 5A09357, 5A09358, 5A09359, 5A0935A, 5A0935B, 5A0935Z, 5A09457, 5A09458, 5A09459, 5A0945A, 5A0945B, 5A0945Z, 0B21XEZ, 5A09557, 5A09558, 5A09559, 5A0955A, 5A0955B, 5A0955Z, 5A1955Z, 5A1945Z, 5A1935Z,  CPT codes available upon request |
